# Fly model sheds light on brain disease

**DOI:** 10.7554/eLife.53233

**Published:** 2019-12-06

**Authors:** Martin H Berryer, Sara G Kosmaczewski, Lindy E Barrett

**Affiliations:** 1 Stanley Center for Psychiatric ResearchBroad Institute of MIT and HarvardCambridgeUnited States; 2 Department of Stem Cell and Regenerative BiologyHarvard UniversityCambridgeUnited States

**Keywords:** cysteine-string protein, neuronal ceroid lipofuscinosis, lysosome, neurodegeneration, *D. melanogaster*

## Abstract

Experiments on flies suggest that a gain-of-function mechanism in a protein called CSPɑ contributes to the progressive brain disease CLN4.

**Related research article** Imler E, Pyon JS, Kindelay S, Torvund M, Zhang YQ, Chandra SS, Zinsmaier KE. 2019. A *Drosophila* model of neuronal ceroid lipofuscinosis CLN4 reveals a hypermorphic gain of function mechanism. *eLife* **8**:e46607. doi: 10.7554/eLife.46607

Neuronal ceroid lipofuscinosis (NCL) refers to a group of progressive brain diseases that affect between 1 and 30 per 100,000 people and are characterized by a decline in motor abilities, seizures, dementia and premature death (Nosková et al., 2011; Haltia, 2003). Most NCLs are caused by the failure of cells to recycle various proteins inside lysosomes (Wisniewski et al., 2001; Henderson et al., 2016; Sambri et al., 2017; Warrier et al., 2013). However, one type of neuronal ceroid lipofuscinosis is different: CLN4 disease occurs when a patient inherits one mutant copy of the gene that encodes for a protein called CSPɑ, but the precise cellular dysfunction underlying CLN4 disease remains a mystery (Nosková et al., 2011; Benitez and Sands, 2017).

Mutations in this gene have been associated with both loss and gain of function. In theory, the mutations could deplete normal CSPɑ, and this loss of function could contribute to disease pathology. Alternatively, the mutations could enhance the normal activity of CSPɑ or lead to an additional, toxic function to drive disease pathology. Now, in eLife, Konrad Zinsmaier and colleagues at the University of Arizona and Yale University – including Elliot Imler as first author – report the generation of a new animal model to investigate the biological mechanisms underlying CLN4 disease (Imler et al., 2019).

Imler et al. started by expressing either the normal or mutant human forms of CSPɑ in flies and confirming that both were functional in fly cells. Next they confirmed that mutant forms of CSPɑ could mimic pathological features seen in CLN4 patients. Interestingly, they found that the severity of the disease correlated with the copy number of the mutated gene: a single copy of the mutated gene did not affect lifespan, but two copies led to more severe phenotypes and early death. To confirm that these results were not an artifact of expressing a human protein in flies, Imler et al. repeated many of their experiments using mutant versions of fly CSPɑ.

The Arizona–Yale team then looked at where mutant CSPɑ resides in neurons. Normally, one would expect to find CSPɑ at nerve terminals, but the mutations resulted in lower levels of the protein at nerve terminals and higher levels in regions of the cell that contained other proteins that had been marked for degradation. By using markers of different cellular components, the researchers demonstrated that mutant CSPɑ was accumulating on prelysosomal endosomes. An endosome is a mini-compartment within a cell that internalizes molecules from the cell membrane: the endosome then fuses with an organelle called a lysosome, and the molecules inside it are broken down and recycled. Imler et al. hypothesize that mutant CSPɑ may be re-routed from nerve terminals through the endolysosomal pathway. Consistent with this, electron microscopy revealed the formation of abnormal membrane structures in cells, which may be due to mutant CSPɑ congesting the trafficking system.

Next, Imler et al. modulated the levels of normal and mutant CSPɑ in cells. Reducing the level of normal CSPɑ reduced the mutant phenotypes, while increasing it exacerbated the mutant phenotypes. Similarly, reducing the level of a heat shock protein that normally interacts with CSPɑ attenuated mutant phenotypes, suggesting that the heat shock protein may play a role in disease biology. The Arizona–Yale team suggests that the disease-causing mutations in CSPɑ enhance its normal activity to disrupt the function of neurons. This gain-of-function mechanism is distinct from that found in the other NCLs, which typically arise from a deficiency in the function of the mutated gene.

The results of Imler et al. demonstrate the value of the fruit fly model to study CLN4 disease pathology. However, these results also paint a complex picture of CLN4, and much work remains to be done to understand how each molecular or cellular pathology contributes to the devastating cognitive and motor deficits found in patients. Future work can now focus on how these biochemical disruptions impact neuronal function over time, taking advantage of the many tools and techniques available in fly genetics in order to interrogate pathways that have additive or compensatory mechanisms that may someday yield therapeutic potential.
